# 1785. Assessment of Antimicrobial Prescription Trends Based on AWaRe Index Among COVID Patients as part of the Implementation of a Modified Antimicrobial Stewardship Audit for COVID Locations of Restricted Access in a South Indian Tertiary Care Center

**DOI:** 10.1093/ofid/ofac492.1415

**Published:** 2022-12-15

**Authors:** T S Dipu, Kiran G Kulirankal, Ann Mary, Fabia Edathadathil, Jina Raj, Merlin Moni

**Affiliations:** Amrita Institute of Medical Sciences and Research Center, kochi, Kerala, India; Amrita Institute of Medical Sciences and Research Center, kochi, Kerala, India; Amrita Institute of Medical Sciences and Research Center, kochi, Kerala, India; Amrita Institute of Medical Sciences and Research Center, kochi, Kerala, India; Amrita Institute of Medical Sciences and Research Center, kochi, Kerala, India; Amrita Institute of Medical Sciences, Kochi, Kochi, Kerala, India

## Abstract

**Background:**

The advent of COVID pandemic disrupted routine antimicrobial stewardship(ASP) activities. We implemented a modified ASP audit in COVID locations using electronic stewardship forms inorder to monitor all antibiotics prescribed to COVID patients and assess them based on WHO AWaRe index.

**Figure:**
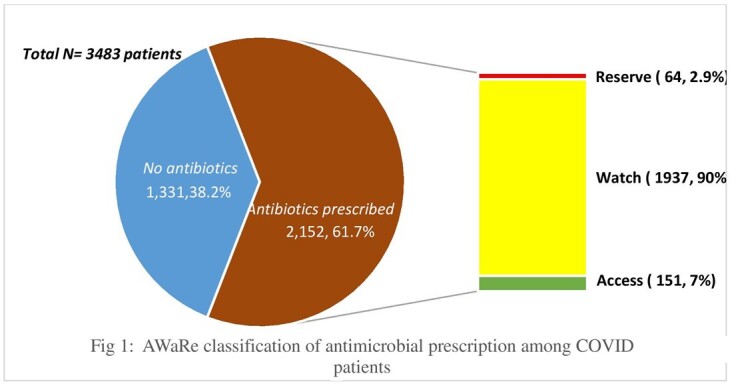

**Figure:**
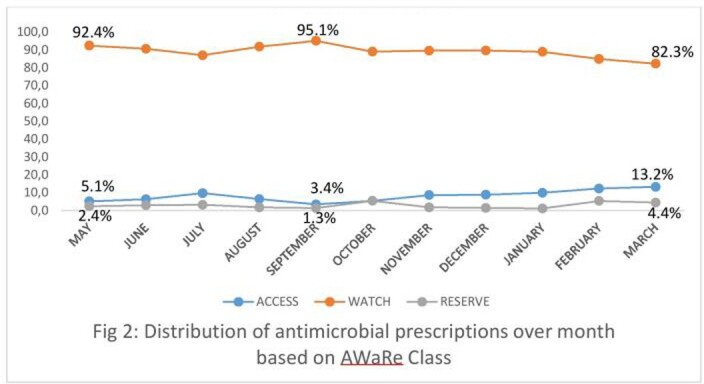

**Methods:**

Our prospective audit included all COVID inpatients who received antimicrobial prescriptions in 70 bedded covid area during the period from May 1^st^, 2021 to March 31st, 2022. All antimicrobial prescriptions were tracked using the daily review of the pharmacy consumption charts in Covid locations and prescriptions were categorised as per AWaRe index. For all reserve antimicrobial prescriptions link for REDCap based E – stewardship forms were sent to the physician in charge of the covid locations.

**Figure:**
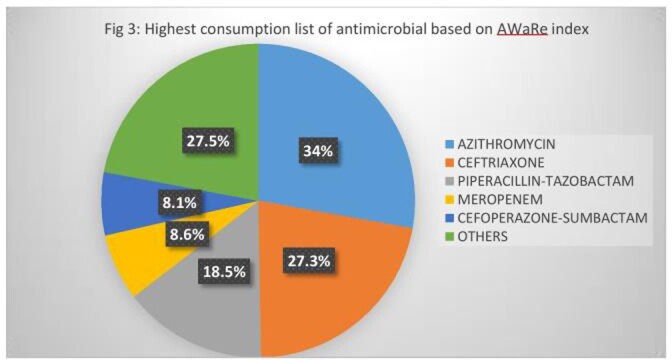

**Results:**

Among 3484 COVID patients reviewed from May 1^st^ 2021 to March 31^st^ 2022, 2152 (61.7%) patients were prescribed with antimicrobials. The highest consumption of antimicrobials prescribed belonged to Watch group( 1937/2152, 90%) followed by Access (151/2152, 7.2%) and Reserve categories (64/2152, 3%) of WHO AWaRe index. Among the Watch group, the highest consumption was observed for Azithromycin (748/2152, 34.7%), followed by ceftriaxone(589/2152,27.3%) , piperacillin- tazobactam(400/2152,18.5%) and Meropenem(187/2152, 8.6%). Linezolid (22/2152, 1.0%) accounted for highest consumption among Reserve group, followed by Ceftazidime –Avibactam (17/2152, 0.7%) and colistin(14/2152, 0.6%).

**Conclusion:**

The study successfully demonstrated the feasibility of implementing modified antimicrobial stewardship in Covid locations during the pandemic, especially in presence of unmet need for targeted strategies for driving antimicrobial stewardship

**Disclosures:**

**All Authors**: No reported disclosures.

